# Seven weeks of pectoralis muscle stretching does not induce non‐local effects in dorsiflexion ankle range of motion

**DOI:** 10.1002/ejsc.12061

**Published:** 2024-02-05

**Authors:** Andreas Konrad, Marina Reiner, Josefina Manieu, Josef Fischer, Adrian Schöpflin, Markus Tilp, David George Behm

**Affiliations:** ^1^ Institute of Human Movement Science Sports and Health University of Graz Graz Austria; ^2^ School of Human Kinetics and Recreation Memorial University of Newfoundland St. John's Newfoundland and Labrador Canada

**Keywords:** crossover, flexibility, global effects, static stretching, stretch training

## Abstract

Both an acute bout, as well as chronic static stretching (SS), can increase the joint range of motion (ROM). However, ROM increases of a non‐stretched muscle (non‐local) are reported following an acute SS session, and these effects have not been studied for long‐term SS training. Therefore, this study aimed to investigate the effects of a comprehensive 7‐week SS training program of the pectoralis muscles on ankle dorsiflexion ROM. Thirty‐three healthy, physically active participants (20 male and 13 female) were assigned to either the SS (*n* = 18) or the control (*n* = 15) group. The SS group performed a 7‐week SS intervention that comprised three sessions a week, including three exercises of the pectoralis muscles for 5‐min each. Before and after the intervention period, the ankle dorsiflexion ROM was tested with a dynamometer. There was no significant time (*p* = 0.93, F_1,31_ = 0.008; *η*2 = 0.000) or time x group effect (*p* = 0. 56, F_1,31_ = 0.342; *η*2 = 0.011) in ankle dorsiflexion ROM, indicating no changes in ROM in the intervention as well as the control group. Although previous studies on the acute effects of stretching reported non‐local increases in ROM, our study showed no such changes after 7 weeks of SS training. Consequently, if the goal is to chronically increase the ROM of a specific joint, it is recommended to directly stretch the muscles of interest.

## Highlights

- This is the first study that investigated the chronic non‐local effects of stretching.
- The results showed no significant changes in the range of motion in the non‐stretched tissues.
- If the goal is to chronically increase the ROM of a specific joint by stretching, it is recommended to directly stretch the muscles of interest.

## 1 INTRODUCTION

Stretching, with all its various techniques, can increase the range of motion (ROM) of a joint acutely following a single bout of stretching (Behm, Blazevich, et al., 2016, Behm, Kay, et al., 2021; Konrad et al., 2017), as well as chronically following stretch training for several weeks (Longo et al., 2021; Mahieu et al., 2007, 2009; Thomas et al., 2018).

Remarkably, a single static stretching exercise not only induces a change in the ROM of the targeted joint but can also impact other non‐adjacent joints, leading to increased flexibility (Behm, Alizadeh, et al., 2021). Various studies have reported acute increases in the ROM of the contralateral homologous (same muscle) limb following a single stretching exercise of the ipsilateral limb (Behm, Alizadeh, et al., 2021; Chaouachi et al., 2017; De‐la‐Cruz‐Torres et al., 2021; Killen et al., 2019). Besides these so‐called contralateral or crossover effects, Wilke et al. (2016) reported an acute increase in cervical ROM (i.e., flexion and extension) following a single static stretching exercise of the gastrocnemius and hamstring muscles (heterologous or dissimilar muscle or area effects). Similarly, Behm, Cavanaugh, et al. (2016) reported acute increases in upper body ROM following a single static stretching exercise of the lower body as well as lower body ROM increases following upper body static stretching. These heterologous changes in the ROM of non‐stretched body regions were mainly attributed to an increased global stretch tolerance (Behm, Alizadeh, et al., 2021).

While the acute effects on non‐stretched body regions have been shown following a single bout of stretching (Behm, Alizadeh, et al., 2021), as well as homologous cross‐education stretch training effects (Moltubakk et al., 2021; Nakamura et al., 2022; Panidi et al., 2021), to the authors' best knowledge, there is no evidence of a chronic change in ROM in other heterologous regions following stretch training of a target muscle group (e.g., stretching the upper body and testing for ROM changes in the lower body).

Therefore, the aim of this study was to investigate the effects of a comprehensive 7‐week static stretching program for the pectoralis muscles, performed 3‐times per week for 15‐min per session, on ankle dorsiflexion ROM. Based on the limited literature examining non‐local heterologous muscle ROM effects following stretching combined with foam rolling (Konrad et al., 2023), we hypothesized that the static stretching program of the pectoralis major would not induce significant changes in ankle dorsiflexion ROM.

## 2 MATERIALS AND METHODS

Participants visited the laboratory on three occasions: for a familiarization session, a pre‐intervention session (pre‐), and a post‐intervention session (post‐) after the 7‐week intervention period. In the pre‐session, the participants were assigned to either the intervention or control (intervention group *n* = 18, control group *n* = 15). Each appointment started with a 5‐min warm‐up on a stationary bike (Monark, Ergomedic 874 E, Sweden) at 60 rev. min‐1 and 60 W. The measurements were performed on the dominant leg (i.e., the leg used for kicking a ball), and the ankle dorsiflexion ROM was assessed. The ethical committee of the University of Graz (approval code GZ. 39/4/63 ex 2021/22) approved the study, and it was conducted in accordance with the standards of the Declaration of Helsinki.

### 2.1 Participants

Although no similar study approach exists, in a previous study, a significant contralateral effect of the dorsiflexion ankle ROM was seen following a static stretch training program on the triceps surae muscle with a large effect size (Cohen's *d* = 1.58) (Panidi et al., 2021). Hence, with the power to detect a large effect size, we calculated a minimum sample size of 15 participants for each group for this study (difference between two dependent means, effect size = 0.8, *α* = 0.05, 1−*β* = 0.8) using G*Power software (Faul et al., 2009).

In total, 33 healthy, physically active volunteers (males: 20, age: 26.4 ± 3.2 years, height: 182.8 ± 9.1 cm, and body mass: 82.1 ± 11.7 kg; females: 13, age: 26.3 ± 4.5 years, height: 165.2 ± 5.9 cm, and body mass: 60.9 ± 9.1 kg) participated in this study. Participants performed different recreational sports such as strength training, soccer, or endurance training with a weekly volume of 9.1 ± 4.0 h. At the familiarization session, the investigator checked the participants' health status with various standardized questions. All the participants confirmed that they had no current musculoskeletal pain or other orthopedic conditions in the lower and upper extremities as well as other non‐specific musculoskeletal disorders (e.g., fibromyalgia). There was no history of surgery or other orthopedic injury in the back, lower or upper extremities in the last 12 months and participants confirmed that there was no neurological disorder, no metabolic disorder, and they took no medication that affects perception or proprioception. Participants were instructed to avoid exhausting training sessions in the 24 h before the measurements. All the procedures were explained to the participants, and each participant gave written informed consent before they were included in the study.

### 2.2 Dorsiflexion range of motion (ROM)

For the dorsiflexion ROM testing, the participant was positioned prone with the ankle fixed on a dynamometer (Con Trex MJ, CMV AG, Dübendorf, Switzerland). The ankle joint axis of the dominant leg was aligned with the rotation axis of the dynamometer using a custom‐made laser device, and the foot was fixed to the footplate with straps. The exact position was noted during the familiarization session, in order to measure the participant in the same position for all appointments. Moreover, to avoid any evasive movements, the participant's trunk was fixed with two “seat belt‐like” straps and an additional fixation was wrapped around the hip. Operating a remote control, the participant was asked to move their ankle joint with an angular velocity of maximum 5°/s into the maximum possible dorsiflexion position (i.e., until the point of discomfort). The maximum position was maintained for a second, and the ankle joint was then moved back into neutral position. This procedure was performed three times, with a 5‐s break between each attempt. The highest ROM value (i.e., the highest dorsiflexion ankle angle) attained was recorded for further analysis. According to a previous study with the same setup in 18 participants (Reiner et al., 2021) the ICC values in ankle ROM between two test days was 0.896 with a 95% confidence interval of 0.719–0.961, and a standard error of the measurement of 2.25°.

### 2.3 Static stretching intervention

**FIGURE 1:**
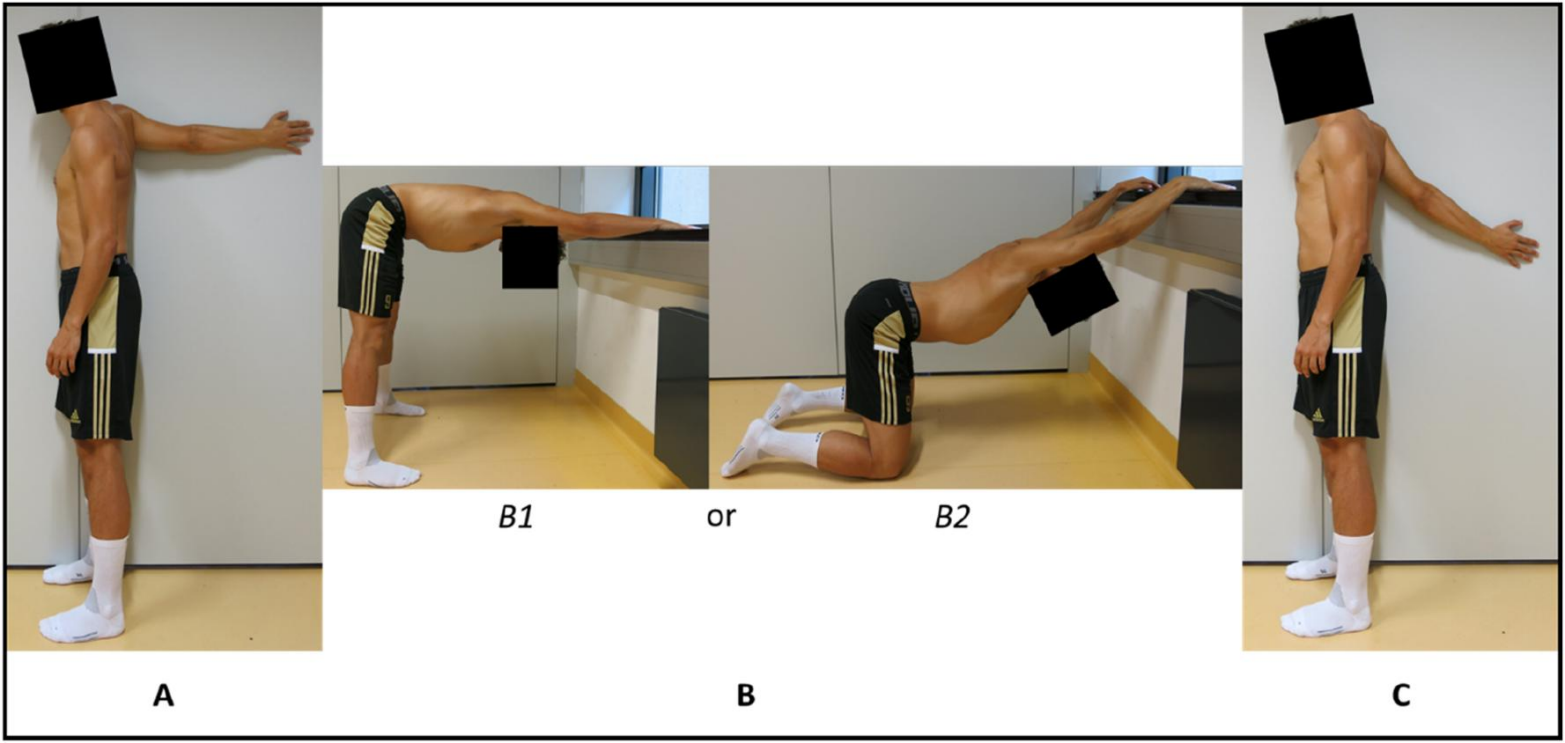
Schematic presentation of the static stretching exercises for the pectoralis muscles. (A) Stretching exercise 1; (B) stretching exercise 2; (C) stretching exercise 3.

The participants of the stretching group were asked to perform the static stretching training, consisting of three exercises for 7‐week, three times a week (recommendation: Monday, Wednesday, Friday). Each exercise was performed continuously for 5‐min, resulting in an overall stretch duration of 15‐min per session. A continuous stretching approach rather than an approach with rest intervals was chosen as Freitas et al. (2015) reported that no rest intervals between the stretches are more effective for increasing the ROM of a joint compared with rest intervals. The stretching exercises were coordinated by a physiotherapist and selected to target the pectoralis muscles. For stretching exercise 1 (Figure 1A), the extended arm was positioned at a wall at shoulder height behind the body, with the palm facing the wall. The stretching intensity was adjusted by rotating the trunk transversely, toward or away from the wall. Stretching exercise 2 was a bilateral stretching exercise, where the hands were placed between breast and hip height on the wall or on an object. The hip was flexed, the arms were kept extended, and the shoulders were moved downwards to the floor (Figure 1B). For increased comfort, the participant was allowed to perform the exercise in a kneeling position (Figure 1B). Stretching exercise 3 was performed similarly to stretching exercise 1, with the difference being that the arm was held at an angle of 45° in relation to the ground, with the palm facing the wall (Figure 1C). For all three stretching exercises, the participant was instructed to maintain the stretch intensity at the point of discomfort during the whole stretch duration. Stretching exercises A and C were performed with the dominant arm, but the participants had the choice to do these exercises bilaterally. In addition to the participant's training diary for training documentation, the stretching exercises were supervised and monitored by an investigator. At least 80% of the training sessions during the 7‐week intervention phase had to be completed by the participants of the stretching group.

Participants of the control group were asked to perform no additional stretching exercises to their normal training routine throughout the intervention period.

### 2.4 Statistical analysis

The statistical analysis was performed using SPSS (version 28, SPSS Inc., Chicago, Illinois). Normal distribution of ROM was confirmed by the Shapiro‐Wilk test. Thus, a mixed model ANOVA (within factor: time (pre‐ vs. post‐test) and between factor: group (intervention vs. control)) was calculated. Effect size partial eta square (η2) was defined as small, medium, and large for effect sizes greater than 0.01, 0.06, and 0.14, respectively (Cohen, 1988). The alpha level was set to 0.05.

## 3 RESULTS

**FIGURE 2:**
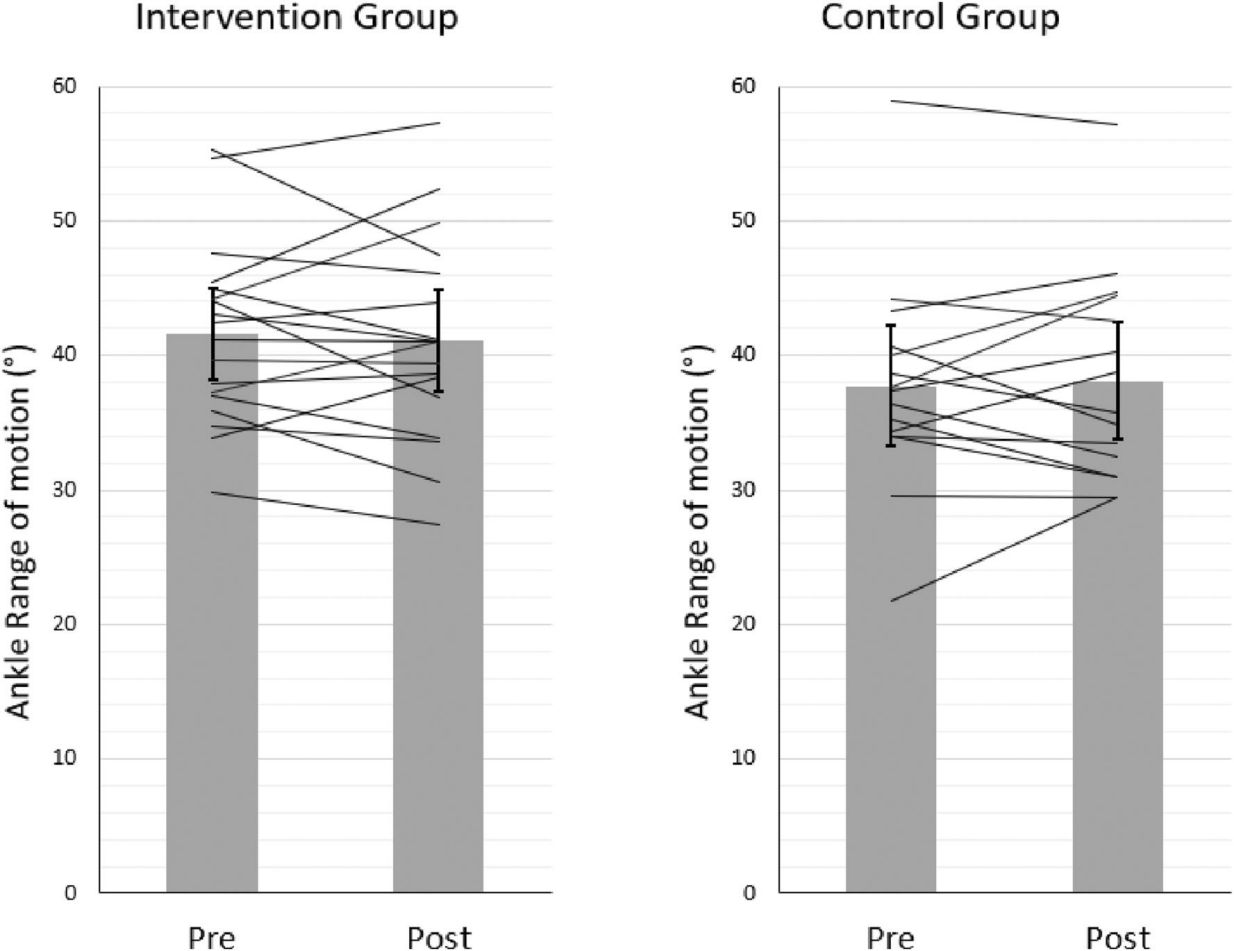
Pre‐ and post‐training mean ROM of both groups (intervention and control groups). Please note that no significant change was observed from pre to post.

ANOVA revealed no significant time (*p* = 0.93, F_1,31_ = 0.008; *η*2 = 0.000) or time x group effect (*p* = 0.56, F_1,31_ = 0.342; *η*2 = 0.011). The ROM values changed during the observation period in the intervention/control group from 41.61 ± 6.73°/37.73 ± 8.01° to 41.11 ± 7.60°/38.09 ± 7.85°, respectively. The individual values are represented in Figure 2.

## 4 DISCUSSION

The aim of this study was to investigate the effects of a comprehensive 7‐week static stretching program of the pectoralis muscles on ankle dorsiflexion ROM. The results showed no significant change in ankle ROM following the upper body stretch program. Indeed, the applied stretching stimulus of the pectoralis muscles is enough to induce local changes in ROM (i.e., shoulder extension) as have been shown in a previous study (Reiner et al., 2023).

A recent meta‐analysis on the non‐local effects of single passive stretches reported acute changes in ROM even in non‐stretched homologous as well as heterologous joints (Behm, Alizadeh, et al., 2021). Although not significant, longer stretch durations (>240 s) per stretching exercise showed large magnitude effect sizes (ES = 1.24) compared to the moderate magnitude increases with shorter stretching durations (<120 s; ES = 0.72). However, according to the subgroup analyses; sex, trained state, or stretch intensity seems to play no role for the magnitude of the acute effect on non‐local increases in ROM (Behm, Alizadeh, et al., 2021). Wilke et al. (2016) reported an acute increase in cervical flexion and extension ROM following a single static stretching exercise of the gastrocnemius and hamstrings muscles. These authors argued that the non‐local increase in ROM might be related to a strain transfer along myofascial meridians. Additionally, Behm, Cavanaugh, et al. (2016) reported acute increases in upper body ROM following a single static stretching exercise of the lower body as well as lower body ROM increases following upper body static stretching. These authors concluded that an increased stretch tolerance on a global level was responsible for the non‐local increases in ROM. Besides that, various studies have found acute increases in ROM of the contralateral limb following a single stretching exercise of the ipsilateral limb (Behm, Alizadeh, et al., 2021; Chaouachi et al., 2017; De‐la‐Cruz‐Torres et al., 2021; Killen et al., 2019) and again this was mainly attributed to an increased global stretch tolerance. It can be concluded that there is vast evidence that a single stretching exercise can increase ROM of non‐stretched body regions acutely, either due to strain transfer along myofascial chains or more likely due to an increased global pain perception.

Taking into account the chronic non‐local effects of stretching on ROM, it is essential to note that current research has primarily focused on investigating the contralateral homologous effects of stretching. Various studies reported an increase in ankle dorsiflexion ROM of the contralateral limb following static stretch training of the ipsilateral limb for several weeks (Moltubakk et al., 2021; Nakamura et al., 2022; Panidi et al., 2021). According to the study of Nakamura et al. (2022) the assessed contralateral effect was attributed to an increase in stretch tolerance rather than changes in muscle stiffness. As seen in recent meta‐analyses, it is evident that long‐term stretch training can locally (i.e., in the stretched muscle) decrease muscle stiffness (Takeuchi et al., 2023) and increase fascicle length (Panidi et al., 2023). However, if no stretch stimulus is applied on the contralateral limb, such a change in muscle structure as seen in the stretched limb is unlikely and rather neurological adaptations may occur in the contralateral limb. Besides the chronic contralateral homologous effects of stretching not much is known about non‐local heterologous changes in ROM following stretch training. One recent study (Konrad et al., 2023) performed a 7 week stretch and foam rolling treatment of the plantar fascia and investigated a potential remote effect on the ankle dorsiflexion ROM. Since foam rolling and stretching seem to have similar acute and chronic effects on ROM (Konrad, Nakamura, & Behm, 2022, Konrad et al., 2023; Wilke et al., 2020) the study of Konrad et al. (2023) can be considered as relevant for our current data. However, Konrad et al. (2023) only found a statistically non‐significant, moderate magnitude (*p* = 0.08, *d* = 0.5) increase in ankle dorsiflexion ROM. Consequently, according to the previous study of Konrad et al. (2023) as well as of the findings in our current study, chronic ROM increases in non‐treated areas (except for homologous contralateral effects) are very unlikely.

This may be related to the cross education “spillover of neural drive” (callosal access) hypothesis whereby adaptations in the control systems in the cortical, subcortical and spinal reflex levels for the trained limb may be accessed by the contralateral limb (Carroll et al., 2006). As there is a homunculi motoneuron organization, the homologous muscles may be spatially closer and thus better able to access this “spillover effect” than the further afield heterologous muscles. With the cross education, cross activation hypothesis, activation of homologous motor networks leads to bilateral activation and adaptations that facilitate subsequent performance (Ruddy & Carson, 2013). In the present context, learning to increase stretch or pain tolerance in one muscle group would be bilaterally taught or transferred preferentially to the contralateral homologous muscle group with less transfer to heterologous muscle groups.

Hence, if the objective is to enhance the ROM of a particular joint, it is advisable to focus on stretching the associated muscle. However, to increase the ROM in the long‐term even other strategies than stretching were reported to increase the ROM to a similar extent. Alizadeh et al. (2023) for example, showed in their meta‐analysis that frequent resistance training performed within the full ROM can chronically increase the ROM of a joint. Besides the increase in ROM, resistance training has certainly other beneficial effects such as for example, increase in muscle strength and muscle mass, reducing back pain, and enhancing cardiovascular health (Westcott, 2012). Moreover, in addition to stretching and resistance training, also frequent foam rolling training can increase the ROM of joints chronically, however, only if performed more than 4 weeks and at specific muscles only (i.e., hamstrings and quadriceps) (Konrad, Nakamura, & Behm, 2022).

This study had some limitations. First, no neuromechanical mechanism such as stretch tolerance or tissue stiffness for potential changes in ROM were assessed in this study. However, since no changes in ROM occurred, it is unlikely that changes in the neuro‐mechanics would have occurred. Second, in this study we included male and female participants. ROM is sex‐dependent with females having greater flexibility than males (Konrad, Bernsteiner, et al., 2022; Medeiros et al., 2013) and this might have led to different stretch‐related adaptations.

## 5 CONCLUSIONS

In conclusion, a 7 week stretch training of the pectoralis muscle, performed for 45 min a week did not induce changes in ankle dorsiflexion ROM. This implies that if the goal is to increase the ROM of a specific joint, a treatment of the associated muscle with either stretching, strength training, or foam rolling is recommended.

## CONFLICT OF INTEREST STATEMENT

The authors report there are no competing interests to declare.

## Data Availability

The data that support the findings of this study are available from the corresponding author upon reasonable request.
